# Live and let live: understanding the temporal drivers and spillovers of life expectancy in Europe for public planning

**DOI:** 10.1007/s10198-022-01469-3

**Published:** 2022-05-26

**Authors:** Pilar Gracia-de-Rentería, Hugo Ferrer-Pérez, Ana Isabel Sanjuán, George Philippidis

**Affiliations:** 1 Agrifood Economics Unit, Agrifood Research and Technology Centre of Aragon (CITA), Avda. Montañana, 930, 50059 Zaragoza, Spain; 2 AgriFood Institute of Aragon–IA2 (CITA—University of Zaragoza), Miguel Servet Street, 177, 50013 Zaragoza, Spain; 3 Aragonese Agency for Research and Development (ARAID), Zaragoza, Spain

**Keywords:** Health production function, Life expectancy, Sustainable development, Spatial panel model, Europe, I12, I15, I14, H51, C33, R12

## Abstract

The European continent has one of the longest life expectancies in the world, but still faces a significant challenge to meet the health targets set by the Sustainable Development Goals of the United Nations for 2030. To improve the understanding of the rationale that guides health outcomes in Europe, this study assesses the direction and magnitude effects of the drivers that contribute to explain life expectancy at birth across 30 European countries for the period 2008–2018 at macro-level. For this purpose, an aggregated health production function is used allowing for spatial effects. The results indicate that an increase in the income level, health expenditure, trade openness, education attainment, or urbanisation might lead to an increase in life expectancy at birth, whereas calories intake or quantity of air pollutants have a negative impact on this health indicator. This implies that health policies should look beyond economic factors and focus also on social and environmental drivers. The results also indicate the existence of significant spillover effects, highlighting the need for coordinated European policies that account for the synergies between countries. Finally, a foresight analysis is conducted to obtain projections for 2030 under different socioeconomic pathways. Results reveal significant differences on longevity projections depending on the adoption, or not, of a more sustainable model of human development and provides valuable insight on the need for anticipatory planning measures to make longer life-spans compatible with the maintenance of the welfare state.

## Introduction

Dating back to the 1920s [1], the demographic transition model has been considered as a seminal framework for population research, employing the five dimensions of birth rates, death rates, natural increases, population size and time to capture four stages of transition. With improvements in life quality over the last century, human development has witnessed rapid declines in death rates (stage 2), as the global population has undergone a “longevity revolution” [2]. Similarly, stage 3 of this paradigm posits significant drops in birth rates. Indeed, a report by the United Nations [3] describes that by 2018 people over 65 outnumbered children under the age of five for the first time, whilst by 2050, the report projects that the over 65 s will constitute a larger group than people aged between 15 and 24.

Focusing on the variable of death rates within the demographic transition model, life expectancy at birth (LEAB) is a key social metric of health. This indicator features prominently in the Sustainable Development Goals (SDGs) under ‘healthy lives and well-being’ [4]. It is one of the indicators used to monitor European progress on SDG 3 by international organizations, such as Eurostat [5] or the Sustainable Development Solutions Network (SDSN) [6]. Moreover, within the United Nations Human Development Index (HDI), life expectancy is considered as one of three fundamental summary metrics for gauging social development [7] as it reflects advances in health care, promotion of responsible and sustainable lifestyles, healthy eating patterns and the development and inclusiveness of other (private and public) social services.

From the perspective of economic policy planning this demographic shift is also of major concern to the welfare state, in particular in developed countries, such as health-care provision and pensions [8, 9]. Moreover, examining the case of the USA, Maestas et al. [10] identify an economic handbrake effect from ageing populations associated with slower growth in the labour force and (more importantly) productivity per worker. In Europe, the Green Deal [11] roadmaps an ambitious vision of prosperity based on the three pillars of sustainability, namely, economy, society and environment. Indeed, through its economic, social and environmental programmes, public policy initiatives also impact on LEAB. Therefore, a clearer understanding of the mechanisms that guide the evolution of LEAB is essential for promoting human well-being but also for anticipatory planning measures, particularly for public finances and social policy.

According to 2018 statistics, the European continent exhibits a LEAB above the world average (79 years and 73 years, respectively) and is second only to North America (80 years) [12]. Moreover, the empirical evidence over the last two decades reveals continued progress in LEAB in Europe, where a child born in 2018 is expected to live 4 years longer than a person born in 2000. Notwithstanding, the rate of this improvement in LEAB has diminished since 2013 due to a slowdown in improvements in some preventable diseases [5].

Within this broadly positive landscape, Europe still faces considerable health challenges related to the issues of unhealthy lifestyles (e.g., obesity, prevalence of smoking) and environmental quality, both resulting in avoidable premature deaths. Taking the former issue, in 2017, 14.9% of the EU population were classified as ‘obese’ and more than half were ‘overweight’, with clear links to the public health risks of widespread chronic diseases (e.g., cardiovascular diseases, diabetes, and certain types of cancer). On the second issue, air pollution is the main environmental cause of death in Europe, being responsible of more than 400,000 premature deaths per year due to respiratory and cardiovascular diseases [5].

There are a number of examples in the literature that examine the impact of economy-wide drivers on LEAB. Most studies focus only on a narrow selection, whilst some consider the linkages between LEAB and the three pillars of sustainability (economic, social and environmental), e.g., [8, 13–16]. With some notable exceptions, however, there is a relative dearth of literature analysing LEAB in Europe [8, 17, 18], where most applications restrict themselves to a handful of countries, thereby ignoring the relative performance of the European continent as a whole and the heterogeneity of outcomes across individual countries.

An issue that has been relatively neglected in the related literature is the possible existence of spatial dependence between regions, which may have consequences in the model misspecification [19, 20]. Spatial dependence refers to the fact that errors can be correlated with errors associated with neighbouring regions. In other words, when the observations are gathered from different regions located in space, it can be often observed that these observations tend to show values similar to those from neighbouring locations instead of being independent in space. Several motivations for this phenomenon are, among others, that the observed variation in the endogenous variable may be influenced by latent unobservable effects related to environmental conditions, lifestyles or culture, the existence of both positive and negative externalities coming from the characteristics of nearby regions or even when economic actors observe past actions of neighbouring actors in their current behaviour [20]. Thus, for example, looking at the adoption of public policy strategies, it may occur that one country can strategically mimic the (health) policies of its neighbours [9, 18], and this may lead to similar strategies and the existence of a ‘spillover’ effect on the (health) indicators of interest. There is a limited literature focusing on spillover impacts for LEAB [9, 14, 21] as well as other health indicators [8, 18, 22–24]. Perhaps surprisingly, despite our observation above that LEAB improves in successive generations, a further trawl through the literature reveals that with the exception of a handful of studies [9, 18, 23, 24], these temporal effects remain under researched, highlighting the need for the construction of a panel data set.

Thus, as a primary aim, this paper revisits the issue of understanding and estimation of the economy-wide determinants of LEAB. To accommodate the temporal element mentioned above, a panel data set is constructed with geographical coverage of the 30 European countries for the period 2008–2018. In particular, we frame our drivers within the tri-dimensional (economy, society and environment) paradigm of sustainability. The econometric model is inspired by the work of Grossman [25] who theorised a ‘health’ production function, whilst further modifications to the econometric specification are implemented to capture spatial dependence effects. In a subsequent step, a series of projections under different socioeconomic pathways for our drivers are implemented into the model to derive the resulting predictive impacts on LEAB in Europe, benchmarking to 2030 in correspondence with the temporal framework of SDGs. The results are discussed with some reflections on the compatibility of sustainable green growth (as per the Green Deal) with desirable changes in the LEAB indicator.

The next section describes the methodological framework. Subsequently, the data and estimation procedure are presented, followed by a presentation and discussion of the results. Employing our results, a foresight exercise to 2030 is presented, followed by the conclusions section.

## Theoretical framework

In the study of Grossman [25], a microeconomic health production function is presented of the form:

1 $$H_{j} = F\left( {X_{j} } \right)$$

where *H*_*j*_ is the health output of individual *j* and *X*_*j*_ is a vector of inputs included in the health production function F. According to a number of commentators [13, 15–17], this microeconomic framework can be scaled up to the macroeconomic level without losing its theoretical grounding by considering per capita or average data, with numerous examples in the recent literature [9, 13–18, 23, 26]. Moreover, macro data analyses have the advantage that the effect of the drivers of LEAB of the overall population can be obtained, providing valuable insights for policy-making. Thus, one can define:

2 $$H = F\left( {Y, S, V} \right)$$

where *H* is the health indicator proxied by LEAB, and *Y*, *S* and *V* are vectors of economic, social and environmental variables, respectively.

Several variables are proposed in the literature to capture the three different driver categories, and in this paper, a selection based on data availability and their relevance in empirical literature is employed. Thus, the economic variables considered are: Gross Domestic Product per capita (GDPpc), health expenditure (both, public and private) and a measure of relative trade openness. The close positive relationship between income level and LEAB is illustrated by the so-called Preston curve [27]. Higher incomes make the consumption of goods and services of higher quality affordable, promoting health [13, 28] and also a better access to health services. Moreover, income level is also found to be correlated with individual behaviours that influence health [29], such as the choice of healthier diets or physical exercise.

The quality of public services is also recognised as a key factor influencing people’s health; an aspect that macroeconomic studies usually proxy through health expenditure data. Importantly, however, a cursory glance at the literature reveals that the effect of this variable remains ambiguous [9, 13, 15, 17, 18, 26, 30]. Indeed, the consensus in the above studies is that the expectation that an increase in health expenditure may improve health services and hence health status, is only true if the marginal effect of this increase is greater than the forgone benefits that would have accrued had these financial resources from taxes been allocated for other purposes with beneficial impacts on health.

Moreover, other studies at macro-level also consider institutional factors, such as globalisation, governance, or corruption, e.g., [31–33]. In the current model specification, a relative openness index is included as a proxy for economic globalization. Once again, however, in the literature there is no clear consensus on the effect of openness on health [34, 35]. On the one hand, openness can benefit health status through the increased trade of medical supplies, drugs and vaccines, the increased mobility of medical staff, technologies and knowledge, and the access to a larger variety of quality food. On the other hand, trade can negatively impact health through (inter alia) the deterioration of working conditions, the transfer of diseases or the adoption of unhealthy consumer practices (e.g., the expansion of fast food).

Turning to the selection of social variables, the level of education, per capita food consumption and income inequality are included. A positive effect of improved education services on life expectancy is widely recognised by international organizations, such as the World Health Organization [32]. In general, people with higher education will be more aware of the importance of health and healthy lifestyles and the potential diseases and cures [13, 15, 28, 36].

The literature also establishes a clear relationship between food and health, since malnutrition in all its forms is shown as a crucial factor influencing LEAB. In general, the macro-level literature has used food availability [13, 15, 16] or caloric deficiency [28, 37] to explore this nexus on developing countries, while fat consumption [18, 36] or obesity [9, 21, 29] are mainly used in studies focused on developed countries. Thus, our maintained hypothesis is that in developed countries, where overconsumption of food is more widespread, further increases in per capita kilocalorie intake and associated obesity problems negatively influence LEAB.

Inequality is a further social factor that can influence LEAB. In short, a more unequal distribution of income is related with higher average poverty levels leading to the inability to cover basic needs, such as housing, food, or basic supplies, therefore, having a negative impact on health [36].

Finally, environmental variables are represented by the per capita pollution level and urbanisation. Air quality is a key conditioning factor of health status [13, 24, 37–39], leading to respiratory and cardiovascular diseases and lung cancer. The relationship between pollution and these diseases is also amply corroborated by the empirical literature, e.g., [23, 40, 41].

The level of urbanisation is another factor considered in the macroeconomic literature, although its effect remains unclear. On the one hand, urbanisation is a proxy of the access to public services [16, 28, 38, 42]. On the other hand, urbanisation can also be associated with congestion and multiple sources of pollution, thereby having an adverse effect on health status [13–15].

## Model, data and estimation

### Model and data

This study is based on the aggregated health production function described in the previous section.^1^ The model specification employs a panel data approach with country fixed effects^2^ that permits a measurement of the relationship between variables after controlling for country heterogeneity:

3 $$\ln {\text{LEAB}}_{it} = \alpha_{0} + X_{it} \beta + \varepsilon_{it}$$

where the subindex *i* represents each of the 30 European countries in the sample^3^ (*i* = 1,…, *N*, where *N* = 30), and *t* = 2008,…, 2018, leading to a total of 330 observations. $LEAB$ is the life expectancy at birth (in log terms to allow non-linear relationships), measured as the number of years a newborn in year *t* is expected to live; ${\alpha }_{0}$ is the constant term; $X$ represents the $TN\times K$ matrix of explanatory variables for economic, social and environmental dimensions that are described in the following paragraphs; $\beta$ is the $K\times 1$ vector of coefficients; and ε denotes the error term.

Regarding the economic drivers, $$\mathit{GDPpc}$$ is the log of GDP per capita (in constant 2010 US$). The variable $$HealthExp$$ is the share of health expenditure in GDP (in %). We opt for this specification, as in [13, 28], rather than health expenditure per capita, as in [15, 17, 26, 30, 37], because the latter may lead to multicollinearity problems due to the high correlation with per capita GDP [13, 28].^4^ The variable $$Openness$$ (in %) is measured as the ratio of the country *i*’s share of trade (exports and imports) on GDP to the world share of trade on GDP.

For the social dimension variables, $School$ is the log of expected years of schooling, defined as the number of years of schooling that a child of school entrance age can expect to receive if prevailing patterns of age-specific enrolment rates persist throughout the child’s life. Aside from its clear definition, this measure is also favoured because of the comprehensive time and country coverage of data, unlike for other education variables usually selected in the previous literature, such as the illiteracy rate, e.g., [13, 15]. The variable $Foodpc$ is the log of daily per capita food consumption (in kcal/capita/day), whilst $Palma$ is the Palma inequality ratio, constructed by dividing the richest 10% of the population’s share of gross national income by the poorest 40% share. Other inequality measures have been also used in the literature, such as the Gini Index [43, 44] or the poverty rate [21, 36]. We opted for the Palma ratio, because it is easy to calculate and reduces oversensitivity to income in the middle of the distribution present in other inequality measures, such as the Gini Index [45]. Moreover, the Palma ratio more faithfully aligns with SDG target 10.1. that states that, “By 2030, progressively achieve and sustain income growth of the bottom 40 per cent of the population at a rate higher than the national average” [4].

With respect to environmental factors, $Pollutpc$ is the log of an aggregated per capita pollution measure (in Kilograms/capita) that includes ozone precursor gases, such as Carbon Monoxide (CO), Nitrogen Oxides (NOx), Non-Methane Volatile Organic Compounds (NMVOC) and Methane (CH4); acidifying gases, such as Ammonia (NH3), Nitrogen oxides (NOx) and Sulfur Dioxide (SO2); and Fine Particulate Matter, as PM10 and PM2.5. Finally, $Urban$ refers to the percentage of urban population.

Data is drawn from the World Bank Development Indicators database [12], except for food consumption that comes from the Food Balances Sheet of the FAOSTAT database [46], the education variable that is taken from the Human Development Data Center [47], and data on air pollutants that is from Eurostat [48].

**Fig. 1:**
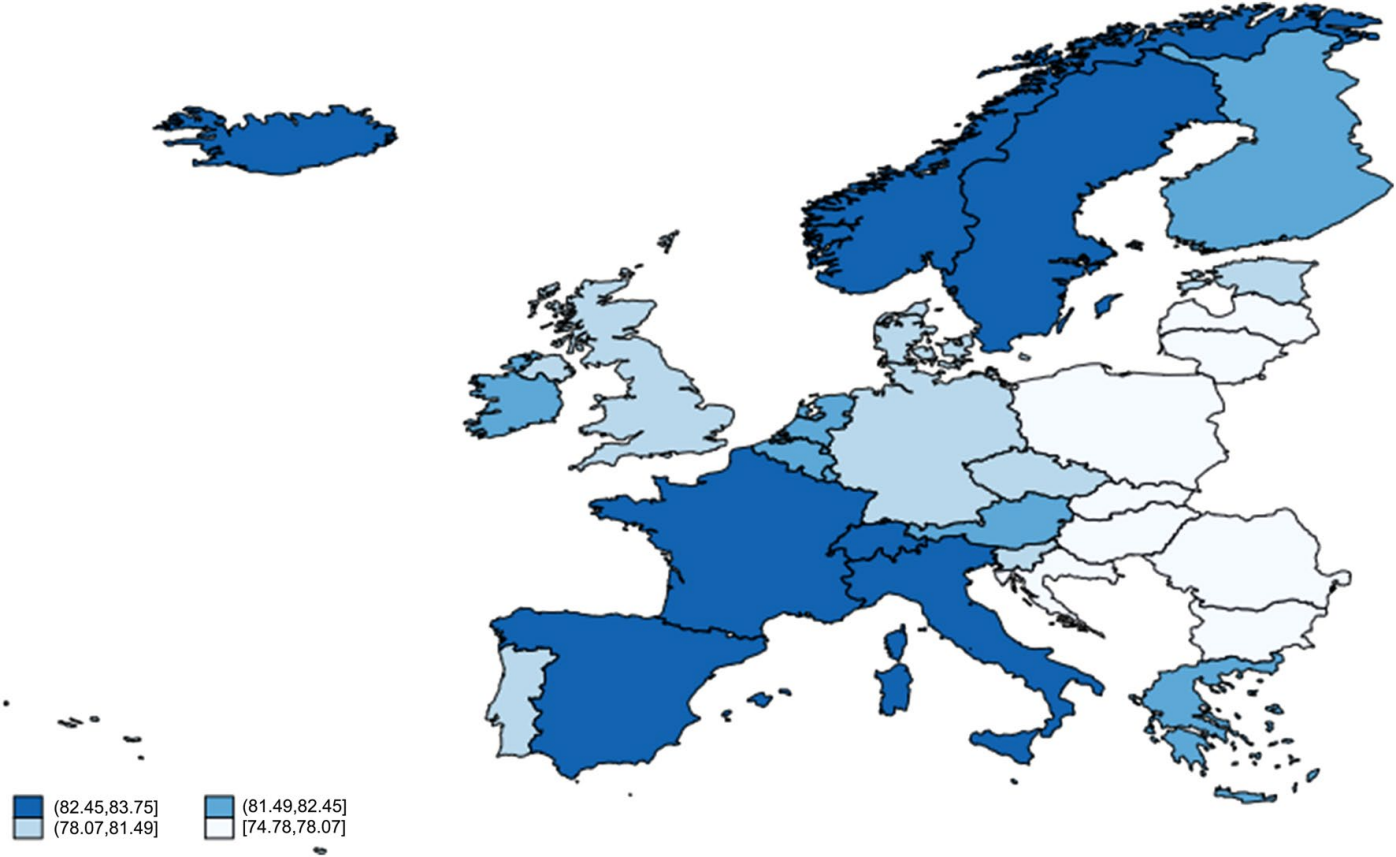
Life expectancy at birth (years) in 2018

Table 1 provides a summary of the descriptive statistics of the variables considered in this study, while Fig. 1 presents the geographical pattern for *LEAB* for the latest year in our sample. From a visual inspection, one can observe the presence of similar values in neighbouring countries that suggest the existence of spatial correlations. The highest *LEAB* values are located in the Mediterranean and Nordic countries, with values between 82.20 and 82.90, whereas Eastern European countries are those with the lowest *LEAB* values, below 77.59 years. Indeed, these outcomes support the dynamics of falling death rates and levels of economic development posited in the demographic transition model discussed previously.

**Table 1:** Descriptive statistics of the explanatory variables

Variable (unit)	Mean	Standard deviation	Min.	Max.
*LEAB* (years)	79.46	2.98	71.81	83.75
*GDPpc* (constant 2010 US$)	37,132.25	23,996.33	6,730.06	110,701.90
*HealthExp* (%)	8.50	1.78	4.70	11.90
*Openness* (ratio)	1.48	0.82	0.65	4.79
*School* (years)	16.48	1.44	13.50	19.80
*Foodpc* (Kcal/person/day)	3,346.36	223.78	2,718.00	3,871.00
*Palma* (ratio)	1.10	0.22	0.67	1.89
*Pollutpc* (Kg/person)	191.73	133.17	53.96	849.98
*Urban* (%)	73.99	12.78	52.21	98.00

### Spatial dependence

To confirm whether the general behaviour of the *LEAB* variable exhibits global spatial autocorrelation, the Moran’s *I* test [49] has been applied to test for the null of spatial randomization, in other words, if data are randomly distributed in space with no spatial associations or clusters. If the test statistic is statistically significant and positive, data show positive autocorrelation with spatial clustering around similar values. That is, nearby countries tend to show similar values of the endogenous variable. If the test is statistically significant but negative, data show negative autocorrelation suggesting dissimilar neighbours.

**Fig. 2:**
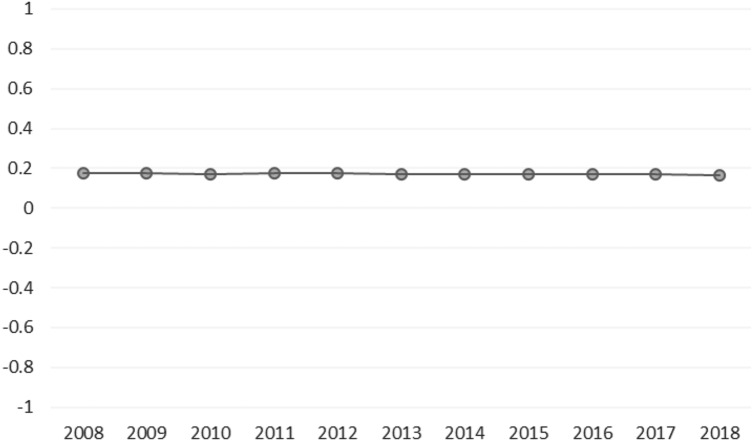
Moran’s *I* test

**Fig. 3:**
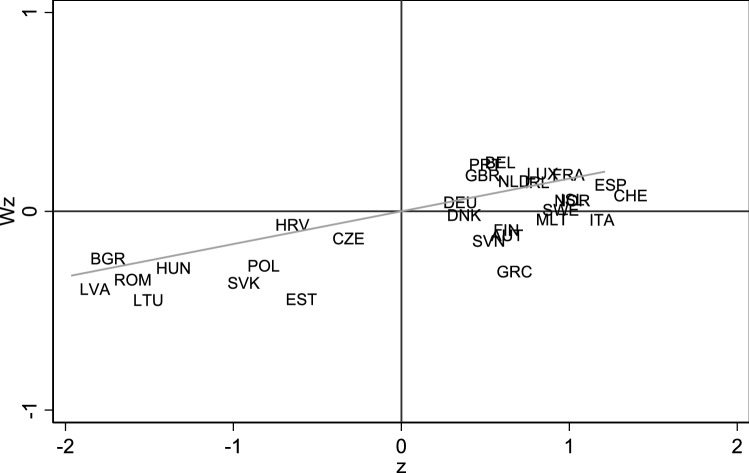
Moran scatterplot for 2018 (Moran’s *I* = 0.165)

Figure 2 presents the application of the Moran’s *I* test for the endogenous variable for each year in the sample period. The results indicate that the null is strongly rejected at 1% significance level suggesting that, in general terms, the *LEAB* variable exhibits spatial dependence. This positive spatial autocorrelation can be also seen in Fig. 3, where the Moran scatterplot [19] for 2018 is used as a measure of local spatial autocorrelation. This scatter plot illustrates the relation between the *LEAB* of each country (horizontal axis) and the average of the *LEAB* of nearby countries (vertical axis), which form the spatial weights matrix W that measures the linkages between the countries. This allows us to detect the existence of spatial clusters, where high values are gathered around high values in neighbouring regions and low values with low values. In particular, in the upper-right quadrant are those countries with *LEAB* values above the mean and that the average of its neighbouring countries is also above the mean, whereas in the lower-left quadrant are located those countries below the mean and that the average of its neighbouring countries is also below the mean. It is noteworthy to mention that there are no countries in the upper-left quadrant, while a few countries have values of life expectancy above the mean but the average of neighbouring countries is below the mean, being placed in the lower-right quadrant.

In view of this exploratory analysis, the presence of spatial dependence in LEAB is confirmed. Therefore, the standard non-spatial model in Eq. (3) may be mis-specified leading to biased results. Consequently, our main hypothesis is that the effect on life expectancy in a certain region is not only influenced by the levels of its respective drivers but also their interdependences across regions.

Although there are alternative estimation procedures available in the literature, a general starting model specification is the Spatial Durbin Panel Model (SDPM), which allows us to estimate the impacts of the drivers on life expectancy as well as their spatial spillover effects across countries. In this sense, the SDPM is particularly suited because of its flexibility to model such spillover effects and might be a useful tool to deal with the potential influence of omitted variables and unobserved heterogeneity [20, 50, 51]. The SDPM model is defined as

4 $$\ln \left( {{\text{LEAB}}_{it} } \right) = \alpha_{0} + \rho W\ln \left( {{\text{LEAB}}_{it} } \right) + X_{it} \beta + WX_{it} \theta + \varepsilon_{it}$$

where $\rho$ is the coefficient associated with the non-negative constant weights $N\times N$ matrix *W*, providing information about the intensity of spatial dependence in the dependent variable. If positive (negative), the presence of complementary (substitution) effects is suggested. $WX$ represents the spatial lag term of the explanatory variables; and $\theta$ is an array of dimension $Kx1$ that contains the parameters that determine the marginal effect of the explanatory variables from neighbouring observations on the life expectancy. Country fixed effects are also included to capture additional country heterogeneity.

A crucial element in Eq. (4) is the weighting matrix $W$ on the spatial connectivity between countries. In this study, the spatial weights matrix *W* is based on distance:

5 $$w_{ij} = \left\{ {\begin{array}{*{20}c} {1/d_{ij} } & {{\text{if}} i \ne j} \\ 0 & {{\text{if}} i = j} \\ \end{array} } \right.$$

where ${d}_{ij}$ is the geographical distance between the (centroids) spatial neighbourhoods. The inverse-distance spatial weights matrix $W$ is assumed to be constant over time and has been normalised to ease the comparison between spatial parameters of the models.

On the whole, one can consider the specification of a SDPM as the starting point, since alternative spatial models, in particular, the spatial lag model (SAR) and the spatial error model (SEM) are nested and can be reduced from the SDPM under certain hypothesis. In particular, from Eq. (4), if we set ${H}_{0}:\theta =0$ and it cannot be rejected then the Durbin model collapses to a SAR model. Moreover, if ${H}_{0}:\theta +\beta \rho = 0$ and holds, the SEM is the appropriate model [52]. Finally, if both hypotheses hold, the non-spatial panel data model is the correct specification. However, if both hypothesis are rejected, then the SDPM is the model that best fits. In our case, the Wald tests^5^ indicate that the two aforementioned null hypotheses should be rejected, indicating that the spatial panel Durbin model best describes our data.

## Results

Table 2 presents the results of the estimation. The second and third columns show the estimated coefficients of the SDPM model obtained using a maximum likelihood procedure, as well as the endogenous and exogenous interaction effects. The last column depicts the results for the non-spatial OLS panel data model. The bottom rows of the table present the spatial rho, as well as the usual goodness of fit measure (*R*^2^), together with the number of observations employed in the estimation. *ρ* = 0.63, which is a highly significant spillover effect that implies that life expectancy in a certain country will increase, on average, around 6% in that country if there is an increase of 10% in the life expectancy of its neighbouring countries.

**Table 2:** Results of estimation

	SDPM		Non spatial panel data
	Main	*W*	Coefficient
GDPpc	0.0333^***^ (0.0043)	0.0709^***^ (0.0221)	0.0506^***^ (0.0100)
HealthExp	0.0028 (0.0037)	0.1409^***^ (0.0244)	0.0078 (0.0071)
Openness	0.0156^***^ (0.0044)	0.1822^***^ (0.0441)	0.0301^***^ (0.0103)
School	0.0189^**^ (0.0084)	0.1911^***^ (0.0594)	0.0235 (0.0230)
Foodpc	− 0.0583^***^ (0.0147)	− 0.0515 (0.0874)	− 0.0919^**^ (0.0408)
Palma	0.0003 (0.0030)	− 0.0107 (0.0200)	− 0.0022 (0.0099)
Pollutpc	− 0.0155^***^ (0.0039)	0.0272 (0.0216)	− 0.0453^***^ (0.0094)
Urban	0.1229^***^ (0.0268)	− 0.3457 (0.2286)	0.1906^***^ (0.0634)
Spatial rho	0.63^***^ (0.1473)		–
*R*^2^	0.85		0.75
*N*	330		330

*, **, *** indicate statistical significance at the 10%, 5% and 1% level, respectively. Standard errors are in parentheses

Notice that only in the case of the non-spatial OLS model the coefficients in Table 2 can be interpreted as marginal effects or the effect of a change in the explanatory variable on the endogenous variable (i.e., elasticity, given that all variables are in logs). In the spatial model, though, some transformation is required [50]. Based on the selected SDPM model, with inverse distance weights, Table 3 presents the direct and indirect effects. The direct effect measures the impact of a change in an explanatory variable in a particular country on the endogenous variable in the country itself, whereas the indirect (or spillover) effect measures the impact of a change in an explanatory variable in a particular country on the endogenous variable in neighbouring countries. Note that the direct effects obtained for the SDPM are different from the estimated main coefficients in Table 2 due to the feedback effects, that is, the effects that are transmitted to neighbouring countries and back to the country itself again prompting the change.^6^

**Table 3:** Direct and indirect marginal effects based on the coefficients of the SPDM showed in Table 2

	Direct effect	Indirect effect
GDPpc	0.0360^***^ (0.0042)	0.1236^***^ (0.0359)
HealthExp	0.0073^*^ (0.0041)	0.1920^***^ (0.0500)
Openness	0.0217^***^ (0.0050)	0.2582^***^ (0.0728)
School	0.0258^***^ (0.0088)	0.2707^***^ (0.0910)
Foodpc	− 0.0603^***^ (0.0153)	− 0.1121 (0.1155)
Palma	0.0001 (0.0032)	− 0.0126 (0.0279)
Pollutpc	− 0.0150^***^ (0.0041)	0.0255 (0.0316)
Urban	0.1139^***^ (0.0230)	− 0.3601 (0.3184)

^*^, **, *** indicate statistical significance at the 10%, 5% and 1% level, respectively. Standard errors are in parentheses

Focusing on the discussion of the direct and indirect effects estimates, one can observe that the level of GDP per capita has a positive and significant effect in its own-region, indicating that an increase of a 1% in the level of economic development leads to an increase of 0.036% in the LEAB of a certain country. In the non-spatial OLS model, the effect is around 0.05%, indicating that if the spatial component is not incorporated in the model, the GDPpc effect on LEAB may be overestimated. Note as well that, since direct effect is 0.0360 and the coefficient estimated is 0.0333, then the feedback effect is 0.0360–0.0333 = 0.0027. This means that the effect that goes across neighbouring countries and back to the investigated country (feedback effect) seems to be around 7.5% of the direct effect. Meanwhile, the effect spilled from a certain country to neighbouring countries triple the direct effect, indicating that the LEAB is increased by almost 0.12% given a 1% increase in the income level of neighbouring countries. The evidence on the positive relationship between GDPpc and LEAB obtained in this study is consistent with the previous literature [13, 14, 16, 21, 28–30, 33, 34, 36–38, 53, 54].

In terms of the share of health expenses over GDP, the results show that if the expenses raise by 1%, the own-country LEAB increases by a 0.01% and the effect also spread to neighbours (elasticity of 0.19). Despite the reduced effect, the positive sign of this statistically significant coefficient would indicate an efficient allocation of health expenses. Something that is not always found in the previous macroeconomic literature, with some papers obtaining a positive effect [9, 15, 17, 26, 37], but others a negative effect [13, 14, 30], or a non-significant one [16].

Regarding the impact of trade openness, there is a direct positive (with an elasticity around 0.02) in its own-country LEAB and in surrounding countries (with a higher impact around 0.26), whilst the feedback effect rises to 28% of the direct effect. A positive effect of globalization on life expectancy is also found by previous macro-level studies [31, 34, 35], indicating that higher levels of globalization and trade would enhance health outcomes through a greater trade of medical goods, services and knowledge.

As for the impact of the social variables, results show that the estimated years of schooling has a positive direct effect in own-country LEAB (elasticity of 0.0258) and indirect effect (elasticity of 0.2707). Therefore, the feedback effect represents 27% of the direct effect. The positive relationship between education and life expectancy obtained in this study is in line with the previous macroeconomic literature [9, 13–16, 21, 28, 34, 36, 42, 53].

The per capita calories intake has a significant negative impact on LEAB, suggesting that an increase in the recommended daily calorie intake can lead to overweight problems that are a demonstrated risk to health and, hence, to life expectancy. Specifically, an increase of a 1% in calories intake is associated with a decrease in LEAB of almost 0.06% in its own-country, whereas there is a weak feedback effect of almost 3% of the direct effect, which is mainly due to the non-significance of the indirect effect. A similar result was also found by previous macro-level studies focused on developed countries that obtain a negative effect of obesity [21, 29] or of fat consumption [18, 36] on life expectancy.

The other social variable considered (the Palma ratio) has not a statistically significant effect on LEAB.^7^ This unclear effect is in line with the previous literature, where also [21, 36] found a non-significant or ambiguous effect on LEAB at an aggregated level.

In the environmental dimension, results reveal that an increase of the level of air pollutants per capita in a certain country has a negative and strongly statistically significant effect in LEAB in its own area (elasticity of − 0.015), while the indirect effect is not statistically significant. The negative relationship between pollution and health was also confirmed by other authors applying a macro-level approach, such as [17, 29, 39, 54].

For the urban variable, we observe a positive effect on life expectancy at birth. In fact, this is the variable with a higher direct effect (elasticity of 0.1139), whilst the indirect effect is not statistically significant. The previous evidence on this matter is ambiguous, since, although some papers also indicates a positive relationship [16, 28, 38, 42], it is also possible to find evidence supporting the opposite [14, 15, 21] and, to a lesser extent, providing a non-significant relationship [13, 35].

### Foresight and scenario analysis

For the purposes of informing public policy planning, a simple scenario analysis is carried out combining the statistically significant elasticities obtained in the selected model (i.e., direct effects with inverse-distance weights matrix) with projected increases in each driver for the period 2010–2030. The choice of end year 2030 coincides with period of evaluation of the SDG framework. In this way, one gains an insight into the contribution of each driver to the evolution of LEAB over the period.

Information about the projection of drivers comes from diverse OECD databases. In particular, per capita GDP information comes from the OECD long-term baseline projections [55], the share of health expenditure on GDP comes from Lorenzoni et al. [56]; the per capita calories intake is taken from OECD–FAO [57]; and the per capita pollutants measure is obtained dividing air pollutants projections [58] by population projections [55]. To calculate changes in trade openness, the trends on imports and exports for the OECD countries and the world for 2010–2022 [59] are extended up to 2030, assuming a constant annual growth rate. With these data and the OECD projection of GDP for 2010 and 2030 for the two regional groupings (OECD countries and the world), the relative openness index for 2010 and 2030 is calculated as in the “Model and data” section, as well as its growth rate. Finally, as OECD databases do not offer projections for the mean years of schooling and the share of urban population, these data are taken from the Wittgenstein Human Capital Data Explorer (HCDE) database [60] and the UN World Urbanization Prospects [61], respectively.

In addition, the foresight exercise is complemented using the scenarios described in two Shared Socioeconomic Pathways (SSP): SSP1 and SSP2 [62]. The latter is a ‘middle-of-the-road’ pathway that assumes that future trends do not shift markedly from historical patterns,^8^ whilst the former takes a greener vision with a broader emphasis on environmental sustainability and human well-being.^9^ Projected trends for the drivers for each transition pathway are obtained from IIASA [63–65], except for the health expenditure and the trade data, for which it has not been possible to obtain projections by SSP. Moreover, due to information constraints, data for some of the variables by SSP needs to be proxied. For example, the evolution of the education variable is proxied through the evolution of the percentage of population with tertiary education, and the variation in per capita food consumption is measured with the evolution of per capita value of agricultural crops demand. For both databases (OECD and IIASA projections), the expected increase of the drivers for the period 2010–2030 in the countries of the sample is assumed to be the same as the average increase in OECD countries. Table 4 informs about the projected variation of each driver for the period 2010–2030 using the different data sources and scenarios.

**Table 4:** Expected variation of drivers for the period 2010–2030

	OECD projections	SSP1 IIASA projections	SSP2 IIASA projections
GDPpc	33%	38%	34%
HealthExp	2%	–	–
Foodpc	5%	− 7%	3%
Openness	26%	–	–
School	13%	17%	8%
Pollutpc	− 28%	− 55%	− 46%
Urban	5%	8%	6%

As a result of this procedure, we obtain a projection of European LEAB for 2030 with a value of 81.24 for the OECD projections, of 82.77 for SSP1 and of 81.64 for SSP2. As a barometer for comparison, the predictions of this study are found to be highly consistent with those from the HCDE database for the European continent (81.13 years on average). Moreover, the results suggest that from 2010, people in the EU will have a LEAB 2.40 years longer under OECD projections, 2.80 years longer under SSP2 and 3.93 years longer under SSP1. Therefore, the LEAB projections vary according to the scenarios considered, but also based on the methodological approach used. To illustrate the latter, the same projections were obtained using the statistically significant elasticities obtained with the non-spatial model, leading to a LEAB value for 2030 of 82.61 for the OECD projections, of 83.46 for the SSP1 and of 82.24for the SSP2. This suggests that the lack of consideration of the existing spatial dependences may lead to biased estimated results and erroneous conclusions for the evolution of LEAB.

**Fig. 4:**
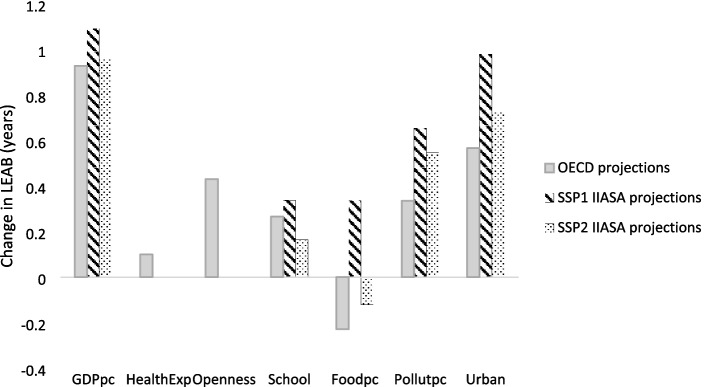
Contribution of each driver to the increase in LEAB between 2030 and 2010 by SSP

To understand the mechanisms behind the differences by socioeconomic pathways, Fig. 4 provides a decomposition of the drivers. First, one can observe that the per capita GDP is the driver with a greatest contribution to the increase in LEAB (which can almost reach a contribution of one more year under the SSP1 for the period 2010–2030), although the other drivers also have a significant contribution. Second, a greater contribution of all the drivers is obtained under the SSP1 than with the SSP2. A particularly different contribution arises for the food consumption variable, which has a negative impact on LEAB under the SSP2 (− 0.12 years) but a positive effect if we shift toward a more sustainable and healthy pathway as in SSP1 (+ 0.34 years). In addition, a significant difference in the contribution to LEAB is shown for the education variable, with a contribution in the SSP1 (+ 0.34) that doubles that of the SSP2 (+ 0.16). Note that SSP1 represents a more favourable scenario than SSP2 for the three pillars of sustainability (economic, social and environmental). The well-known trade-off between socioeconomic development and environmental impact is not reflected in these projections, because SSP1 assumes that technological progress and efficiency improvements will help to overcome this trade-off. In addition, SSP1 assumes a lower increase in population than SSP2 (for more details, see [62]), which is reflected in the higher per capita GDP magnitudes (see Table 4).

## Conclusions

This paper aims at assessing the impact of the macroeconomic factors driving LEAB in the European continent, accounting for spatial dependence across countries. Our analysis is built upon an aggregated health production function and estimated using a spatial Durbin panel model, which fits theory and allows us to retrieve the direct impact, the spillover effect and the simultaneous feedback effect of the drivers examined for a panel data set of 30 countries over the period 2008–2018.

Results obtained provide valuable information about the rationale that guides the evolution of this indicator in the context of the SDGs and their economic, social and environmental domains. As expected, economic factors play a key role on health status at macro-level, since we observe a positive and significant effect of GDP per capita, trade openness and, to a lesser extent, of health expenditures on LEAB. However, this study also concludes that policy-makers should look beyond the factors related with the health system and the economic performance. This means that public policies aimed at enhancing health status should also focus on social and environmental conditions. In the social sphere, the results of this study indicate that LEAB is positively influenced by education and negatively influenced by food calories intake, whereas in the environmental sphere a negative relationship between LEAB and air pollutants and a positive relationship with urban population is confirmed.

The policy implications of these results are noteworthy to mention. On the one hand, education policies aimed at increasing participation rates by discouraging early leaving and promoting tertiary education, but also at improving education outcomes, might empower people to adopt more healthy and sustainable lifestyles. In this regard, the European continent has scope for improvement, since more than 22% of 15-year-old pupils in the EU showed insufficient abilities in reading, mathematics and science [5]. On the other hand, European institutions and countries should intensify their efforts to combat obesity, which is claimed as one the most serious health issues in the continent. In this regard, the encouragement of more sustainable diets combined with physical activity and more healthy lifestyles in general is needed, together with the promotion of an improved and wide-spread use of Food Labelling Information Systems and the maintenance of an updated regulation on nutrition and health claims. In the environmental domain, the focus should be put on air pollutants, in the light of its negative effect on LEAB pointed out by this study. Although exposure to air pollution in the EU has decreased more than 14% since 2011, a greater effort should be done, especially in urban populations with a higher concentration of population and economic activity [5], and where a trade-off between greater access to services and congestion could arise.

The results of this study also indicate that life expectancy in a certain region not only depends on its own drivers but also on those of neighbouring countries, highlighting the need for coordinated policies at European level. Public policies aiming at better health status in a country (i.e., through increases in health expenditure, promoting healthy diets and the reduction of calorie intake) can benefit from policy actions taken in nearby countries.

To that end, a number of efforts have been made within the European continent. To name a few, the numerous European initiatives to address obesity problems, such as the Strategy on Nutrition, Overweight and Obesity-related Health Issues [66], or the EU Action Plan on Childhood Obesity 2014–2020 [67]. Other noteworthy initiatives are the increased efforts to reduce air pollution in the European continent, for example, by means of the Clean Air Programme [68], the Directive on emissions of atmospheric pollutants [69] or the recent European Green Deal [11]. In our view, all these sort of efforts should be maintained and strengthened and the SDGs must be the roadmap of the European policies aimed at meeting the economic, social and environmental sustainability objectives needed to improve health and human wellbeing.

In addition, the COVID-19 crisis has laid on the table the need for a greater coordination in health matters to achieve resilient health systems. But also, this crisis has highlighted the importance of strengthening socioeconomic and climate resilience to face possible future threats to human health, prosperity and environmental sustainability [6]. In this context, an interesting line of future research may address to what extent the COVID-19 crisis has conditioned the factors driving LEAB and evaluate if this event has caused either a transitory or permanent change in both LEAB and its drivers.

A clear limitation of this study is the geographical scope as it is restricted to Europe. Extending the analysis to other locations or even conducting a worldwide analysis in future studies would allow a better understanding of underlying life expectancy drivers under diverse levels of development. In a similar way, a detailed analysis of the differences across European regions according to different classifications (i.e., EuroVoc, old and new EU countries) could be a promising future research direction. Other limitations of this paper derive from its macroeconomic approach, what precludes and analysis of intra-country or inter-individuals differences in the response of life expectancy to more detailed determinants, such as individuals’ health-related behaviours. However, this macro-level model informs well on the average impact of selected drivers on the population average of LEAB. In this regard, the consideration of other health proxies could be an extension of this research, in particular the study of the drivers of the ‘healthy life years at birth’ indicator recently proposed by Eurostat to measure progress on health and well-being [70].

Nevertheless, this study provides a suitable comparison framework for the analysis of how alternative socioeconomic pathways may influence the average countries’ longevity. For this purpose, this study also carried out a scenario analysis that shows the significant differences on LEAB at macro-level that may arise if we opt for a more sustainable and human well-being oriented socioeconomic model, instead of continuing with the business as usual pattern. This exercise provides valuable information about the cost of inaction in terms of longevity and, therefore, in human lives, especially in relation to food consumption and associate overweight problems, and to education.

In any case, the projections presented in this study reveal a significant increase in LEAB in the coming years, which seems to be a double-edged sword from the point of view of public policies. On the one hand, in the aggregated health production function approach, LEAB is a health outcome in a model in which health is view as human capital investment. Therefore, increases in longevity would be associated with improvements in the health status of individuals at all ages, thus reducing the pressure on health systems. On the other hand, this greater longevity means a greater number of elderly people, which may imply a higher demand for health care services, lower work productivity and, therefore, greater pressure on health and pensions systems. This poses important challenges in terms of future resource allocation and budget planning decisions, so Governments and EU institutions should anticipate the necessary planning measures to make greater longevity compatible with the maintenance of the welfare state.

## Footnotes

1 The study has been performed using Stata 17 MP.

2 The Hausman’s specification test supports the use of a fixed effects model (Statistics value = 63.94 and *p* value = 0.000).

3 The 30 European countries included in this study are Austria, Belgium, Bulgaria, Croatia, Czechia, Denmark, Estonia, Finland, France, Germany, Greece, Hungary, Iceland, Ireland, Italy, Latvia, Lithuania, Luxembourg, Malta, Netherlands, Norway, Poland, Portugal, Romania, Slovakia, Slovenia, Spain, Sweden, Switzerland, and the United Kingdom. All European countries with enough information to construct a balanced panel dataset were considered.

4 In fact, using the share of health expenditure in GDP reduces the high correlation coefficient between per capita health expenditure and per capita GDP from 0.928 to 0.425.

5 The statistic value for ${H}_{0}:\theta =0$ is 54.78 (*p* value = 0.000) and the statistic value for ${H}_{0}:\theta +\beta \rho = 0$ is 50.25 (p-value = 0.000).

6 See Elhorst [50, pp. 22–23, 31] for detailed calculations of direct, indirect and feedback effects.

7 A similar result is obtained when the model is re-estimated using the Gini coefficient instead of the Palma ratio as the inequality measure.

8 Specifically, SSP1 is defined as: “The world shifts gradually, but pervasively, toward a more sustainable path, emphasizing more inclusive development that respects perceived environmental boundaries. Management of the global commons slowly improves, educational and health investments accelerate the demographic transition, and the emphasis on economic growth shifts toward a broader emphasis on human well-being. Driven by an increasing commitment to achieving development goals, inequality is reduced both across and within countries. Consumption is oriented toward low material growth and lower resource and energy intensity” [62].

9 SSP2 is defined as: “The world follows a path in which social, economic, and technological trends do not shift markedly from historical patterns. Development and income growth proceeds unevenly, with some countries making relatively good progress while others fall short of expectations. Global and national institutions work toward but make slow progress in achieving sustainable development goals. Environmental systems experience degradation, although there are some improvements and overall the intensity of resource and energy use declines. Global population growth is moderate and levels off in the second half of the century. Income inequality persists or improves only slowly and challenges to reducing vulnerability to societal and environmental changes remain” [62].
